# Supplementary material for: Clinical Pharmacists’ Knowledge of and Attitudes toward Pharmacogenomic Testing in China
Source: J Pers Med. 2022 Aug 21;12(8):1348. doi: 10.3390/jpm12081348 (PMC9410027; doi:10.3390/jpm12081348)
Supplement: Supplementary file 1 [file jpm-12-01348-s001.zip › jpm-1802820-supplementary.pdf]

Journal of Personalized Medicine

MDPI

## Supplementary Materials

**Figure S1.** Distribution of province and department. (N=1005)

| Province \ Department | Card | Onco | Resp | ICU | Cere/ Neur | Pedi | Dige | Surg | Endo | O&G | Neph | Hema | Infe | Psy | Pain | Emer | Rheu | Derm | Others | Total |
|---|---|---|---|---|---|---|---|---|---|---|---|---|---|---|---|---|---|---|---|---|
| ZJ | 5 | 14 | 8 | 8 | 7 | 3 | 2 | 2 | 0 | 13 | 2 | 4 | 2 | 0 | 2 | 1 | 1 | 0 | 7 | 81 |
| GX | 9 | 6 | 12 | 6 | 5 | 7 | 8 | 5 | 4 | 2 | 4 | 2 | 2 | 0 | 0 | 0 | 0 | 0 | 7 | 79 |
| HeN | 11 | 7 | 16 | 4 | 5 | 4 | 3 | 0 | 8 | 3 | 2 | 0 | 0 | 1 | 2 | 0 | 0 | 1 | 6 | 73 |
| HLJ | 13 | 9 | 10 | 5 | 9 | 4 | 3 | 3 | 3 | 1 | 0 | 0 | 0 | 1 | 0 | 1 | 0 | 0 | 3 | 65 |
| JS | 5 | 6 | 6 | 7 | 4 | 5 | 3 | 1 | 3 | 5 | 3 | 2 | 2 | 1 | 1 | 0 | 1 | 0 | 4 | 59 |
| SC | 7 | 6 | 3 | 3 | 2 | 3 | 1 | 9 | 2 | 5 | 0 | 1 | 2 | 0 | 0 | 0 | 0 | 0 | 5 | 49 |
| CQ | 5 | 11 | 3 | 3 | 2 | 2 | 1 | 4 | 1 | 4 | 3 | 0 | 0 | 0 | 0 | 1 | 2 | 0 | 4 | 46 |
| GS | 3 | 10 | 7 | 4 | 3 | 0 | 4 | 1 | 1 | 0 | 0 | 1 | 1 | 1 | 0 | 0 | 0 | 0 | 5 | 41 |
| JX | 9 | 3 | 4 | 5 | 2 | 3 | 1 | 1 | 4 | 1 | 1 | 2 | 1 | 1 | 1 | 0 | 0 | 0 | 2 | 41 |
| BJ | 3 | 1 | 4 | 1 | 2 | 5 | 2 | 3 | 0 | 4 | 2 | 1 | 1 | 1 | 1 | 2 | 0 | 0 | 1 | 34 |
| SH | 3 | 3 | 4 | 2 | 3 | 1 | 3 | 0 | 3 | 2 | 0 | 1 | 1 | 6 | 0 | 1 | 0 | 0 | 1 | 34 |
| JL | 8 | 3 | 3 | 4 | 2 | 0 | 2 | 4 | 1 | 0 | 1 | 0 | 0 | 0 | 0 | 0 | 0 | 0 | 2 | 30 |
| S3X | 4 | 3 | 5 | 2 | 1 | 2 | 2 | 1 | 1 | 2 | 1 | 0 | 0 | 0 | 0 | 0 | 1 | 0 | 2 | 27 |
| HuN | 4 | 6 | 1 | 3 | 2 | 1 | 0 | 1 | 1 | 1 | 1 | 1 | 0 | 0 | 1 | 0 | 0 | 0 | 3 | 26 |
| SD | 6 | 4 | 3 | 2 | 2 | 4 | 1 | 1 | 1 | 0 | 0 | 1 | 0 | 0 | 1 | 0 | 0 | 0 | 0 | 26 |
| GZ | 2 | 1 | 5 | 1 | 0 | 3 | 4 | 1 | 1 | 0 | 1 | 1 | 1 | 0 | 0 | 0 | 0 | 0 | 4 | 25 |
| XJ | 3 | 7 | 3 | 2 | 0 | 0 | 0 | 2 | 1 | 0 | 2 | 1 | 1 | 0 | 1 | 0 | 1 | 0 | 1 | 25 |
| HeB | 7 | 3 | 1 | 2 | 1 | 3 | 4 | 0 | 1 | 1 | 0 | 0 | 0 | 0 | 0 | 0 | 0 | 0 | 1 | 24 |
| FJ | 0 | 2 | 1 | 2 | 3 | 1 | 1 | 4 | 2 | 0 | 0 | 0 | 1 | 1 | 1 | 0 | 0 | 0 | 3 | 22 |
| YN | 4 | 4 | 0 | 5 | 1 | 3 | 1 | 0 | 0 | 2 | 2 | 0 | 0 | 0 | 0 | 0 | 0 | 0 | 0 | 22 |
| NMG | 3 | 2 | 3 | 3 | 0 | 1 | 1 | 0 | 4 | 1 | 0 | 1 | 1 | 0 | 0 | 0 | 0 | 0 | 1 | 21 |
| HuB | 2 | 2 | 2 | 2 | 3 | 2 | 0 | 3 | 2 | 0 | 1 | 1 | 0 | 0 | 0 | 0 | 0 | 0 | 0 | 20 |
| AH | 4 | 0 | 4 | 1 | 2 | 2 | 4 | 1 | 0 | 0 | 0 | 0 | 0 | 0 | 0 | 0 | 0 | 0 | 0 | 18 |
| GD | 4 | 2 | 1 | 2 | 3 | 2 | 1 | 1 | 0 | 0 | 0 | 0 | 0 | 0 | 0 | 0 | 1 | 0 | 1 | 18 |
| S1X | 2 | 6 | 1 | 0 | 0 | 2 | 0 | 1 | 1 | 0 | 1 | 1 | 0 | 0 | 1 | 0 | 0 | 0 | 2 | 18 |
| TJ | 1 | 5 | 1 | 2 | 0 | 1 | 3 | 2 | 0 | 0 | 1 | 0 | 1 | 0 | 0 | 0 | 0 | 0 | 1 | 18 |
| LN | 1 | 2 | 4 | 1 | 2 | 0 | 2 | 1 | 1 | 0 | 0 | 1 | 0 | 0 | 0 | 1 | 0 | 0 | 0 | 16 |
| NX | 2 | 1 | 0 | 1 | 2 | 1 | 2 | 1 | 2 | 0 | 0 | 0 | 1 | 0 | 0 | 1 | 0 | 0 | 2 | 16 |
| QH | 3 | 2 | 1 | 1 | 0 | 0 | 2 | 1 | 1 | 0 | 2 | 0 | 2 | 1 | 0 | 0 | 0 | 0 | 0 | 16 |
| HaiN | 3 | 0 | 1 | 1 | 1 | 1 | 1 | 1 | 1 | 0 | 1 | 0 | 0 | 0 | 1 | 1 | 0 | 0 | 1 | 14 |
| XZ | 1 | 0 | 0 | 0 | 0 | 0 | 0 | 0 | 0 | 0 | 0 | 0 | 0 | 0 | 0 | 0 | 0 | 0 | 0 | 1 |
| Total | 137 | 131 | 117 | 85 | 69 | 66 | 62 | 55 | 50 | 47 | 31 | 22 | 20 | 14 | 13 | 9 | 7 | 1 | 69 | 1,005 |

Annotation:

Province: Zhejiang (ZJ), Guangxi (GX), Henan (HeN), Heilongjiang (HLJ), Jiangsu (JS), Sichuan (SC), Chongqing (CQ), Gansu (GS), Jiangxi (JX), Beijing (BJ), Shanghai (SH), Jilin (JL), Shaanxi (S3X), Hunan (HuN), Shandong (SD), Guizhou (GZ), Xinjiang (XJ), Hebei (HeB), Fujian (FJ), Yunan (YN), Neimenggu (NMG), Hubei (HuB), Anhui (AH), Guangdong (GD), Shanxi (S1X), Tianjin (TJ), Liaoning (LN), Ningxia (NX), Qinghai (QH), Hainan (HaiN), Xizang (XZ).

Department: Cardiovascular (Card), Oncology (Onco), Respiratory (Resp), Cerebrovascular/ Neurology (Cere/Neur), Pediatrics (Pedi), Digestion (Dige), Surgery (Surg), Endocrine (Endo), Obstetrics and Gynecology (O&G), Nephrology (Neph), Hematology (Hema), Anti-infectives (Infe), Psychiatry (Psy), Emergency (Emer), Rheumatology (Rheu), Dermatology (Derm).
